# An Update on Reports of Atypical Presentations of Kawasaki Disease and the Recognition of IVIG Non-Responder Children

**DOI:** 10.3390/diagnostics13081441

**Published:** 2023-04-17

**Authors:** Cristiano Conte, Francesco Sogni, Donato Rigante, Susanna Esposito

**Affiliations:** 1 Pediatric Clinic, Department of Medicine and Surgery, University Hospital, University of Parma, 43126 Parma, Italy; 2 Department of Life Sciences and Public Health, Fondazione Policlinico Universitario A. Gemelli IRCCS, 00168 Rome, Italy; 3 Università Cattolica Sacro Cuore, 00168 Rome, Italy

**Keywords:** acetylsalicylic acid, coronary artery abnormality, coronary artery aneurysm, innovative biotechnologies, intravenous immunoglobulin, Kawasaki disease, personalized medicine, vascular complications

## Abstract

Kawasaki disease (KD) is an acute vasculitis with an intrinsic risk of severe involvement of coronary arteries. The worldwide spread of KD and the importance of early diagnosis for preventing cardiovascular complications have ascertained the need for updating guidelines for prompt disease recognition and treatment efficacy assessment. All KD patients who comply with the definition of classic or atypical disease should be treated with intravenous immunoglobulin (IVIG) soon after diagnosis. The objective of our narrative review was to analyze the medical literature about case reports with atypical KD in relation to diagnosis and potential identification of predictors of non-responsiveness to IVIG. Our analysis has shown that the seminal challenge in KD management is the timeliness of diagnosis, although both extreme variability and transience of clinical manifestations make this goal difficult. A non-negligible percentage of patients, especially in the first 6 months of life, might have atypical manifestations of KD, whose painstaking differential diagnosis may be tricky. Many attempts to develop universal scoring systems and detect children at higher risk of IVIG resistance have been rather unsuccessful. Additionally, KD may show different evolutions according to unraveled demographic, genetic, or epigenetic factors. Further research is needed to elucidate all open questions about KD and clarify the long-term outcome of its potential complications.

## 1. What We Actually Know about Kawasaki Disease

Kawasaki disease (KD) is an acute childhood vasculitis of small- and medium-sized vessels bearing the risk of severe involvement of coronary arteries, which mainly affects children under 5 years of age. Its tendency of affecting the Asian population to a wider extent has been largely recognized [1]. The worldwide spread of KD and the importance of early diagnosis for preventing its cardiovascular complications have clarified the need for periodic updates of different guidelines aimed at a better definition of KD for prompt disease recognition and assessment of treatment efficacy. The most recent recommendations for KD management are referred to the American Heart Association Guidelines (AHA) of 2017 [2] and Italian Guidelines of 2018, revised in 2021 [3,4,5]. Since the first KD patients were reported in Japan during the past century, this condition has become familiar to Japanese doctors, and many nationwide epidemiologic surveys have been performed through the years. To date, KD has been recognized across all continents, although we know that its incidence in northeast Asian countries, including Japan, South Korea, and Taiwan, is 10–30 times higher than in the United States of America and Europe [6]. In Italy the actual incidence of KD is not known, although an epidemiological study performed by Mauro et al. in 2016 revealed an incidence of 17.6 for 100,000 children under 5 years in the regions of Tuscany and Emilia-Romagna [7]. Cimaz et al. studied the epidemiology of KD in the years 2008–2013 in Italian hospitalized children aged 0–14 years using discharge codes: they found a disease peak in the first 2 years of life in 85.5% of cases under 5 years with an incidence rate of 14.7 per 100,000 children of the same age [8]. Despite over 50 years of clinical observations and in-depth research, the exact etiology of KD remains unknown, although the main pathogenetic disease mechanisms recall a dramatic inflammatory response in genetically predisposed children following a ‘potential’ infectious trigger [9]. Indeed, KD mainly affects children with a likely genetic susceptibility and is nowadays conceived as a systemic multifactorial autoinflammatory disorder [10,11] due to its similarity with hereditary autoinflammatory diseases, which arise from specific dysregulation of innate immunity with no specific age or gender distribution [12,13]. The objective of our research was to review the medical literature about case reports of KD in relation to the diagnosis of atypical disease presentations and the potential identification of predictors of non-response to intravenous immunoglobulin (IVIG). Scientific reports were searched in the PubMed electronic database until December 2022; the keywords used were “Kawasaki disease”, “Kawasaki syndrome”, and “atypical Kawasaki disease”, while articles containing the words “SARS-CoV-2”, “COVID”, and “MIS-C” were excluded by our analysis; additional reports were identified and examined through the specific references cited in the retrieved papers. Over 270 articles were found, but only case reports concerning children diagnosed with atypical KD published in English providing substantial details referring to the objective of our research were included, as well as studies assessing non-responsiveness to IVIG.

## 2. The Conundrum of Kawasaki Disease for Both Diagnosis and Management

Diagnosis of classic KD is clinical and anchored to the presence of high fever persisting for at least 5 days with the presence of at least four of the following five findings: (a) oral changes in terms of mucositis; (b) non-exudative conjunctivitis in both eyes; (c) polymorphous skin rash; (d) abnormalities of the extremities and perineum; and (e) cervical lymph node enlargement, which might be unilateral. Diagnosis can also be established if less than four findings are recognized, but only if coronary artery abnormalities (CAA) are demonstrated on echocardiography. Labwork generally does not show specific changes, but the most commonly found are leukocytosis, neutrophilia, increased erythrocyte sedimentation rate, increased C-reactive protein (CRP), hyponatremia, and hypoalbuminemia [14]. The risk of cardiovascular involvement after KD is a major cause of morbidity and mortality. Coronary arteritis is the first pathological step followed by vascular dilatation as a result of post-inflammatory destruction of vessel internal membranes [15]. KD coronary artery aneurysms display a risk of rupture or thrombosis and may lead to the development of stenotic lesions, which may cause ischemic heart disease evolving to acute myocardial infarction in 0.2–0.5% of cases, even if anti-platelet prophylaxis is initiated [16]. Atypical forms of KD are associated with a higher risk of vascular changes, mainly due to the diagnostic delay and subsequent belated start of treatment [17,18]. Treatment of KD within 10 days after disease onset can lead to abatement of vascular changes, even if a minority of properly treated patients might develop CAA and even giant aneurysms in 1% of cases [2]. Higher values of CRP and younger age at disease onset seem pivotal in determining, respectively, a failure in the response to IVIG and an increased occurrence of CAA [18]. All KD patients who comply with the definition of classic or atypical disease should be treated with IVIG (at a dose of 2 g/kg of body weight) soon after diagnosis [2]. Approximately 10–20% of KD patients do not respond to standard treatment with IVIG, and this peculiar cluster has a higher risk of developing vascular injuries [19]. The intensification of initial therapy or second-line therapies has been a main focus in KD research. Other useful medications for IVIG refractory patients include corticosteroids and biological drugs against the tumor necrosis factor (TNF), such as infliximab and etanercept, or against interleukin (IL)-1, such as anakinra, as plasma levels of both TNF and IL-1 were found elevated in the acute phase of KD [20]. According to more recent guidelines, “IVIG resistance” is defined as the persistence or recurrence of a fever (≥38 °C) from 36 h to 7 days after the completion of IVIG infusion [21]. There are conflicting studies on factors, both clinical and laboratory data at disease onset, that might predict which patients will be IVIG non-responsive, and different risk scores have not been useful for the same goal in ‘all’ KD patients or in those with atypical disease patterns [22,23]. Nevertheless, many randomized controlled trials and observational studies have shown that anti-cytokine biologics do not seem effective in reducing the frequency of CAA, although they might decrease the frequency of treatment resistance in comparison with the conventional IVIG therapy alone [24].

## 3. Atypical Presentations of Kawasaki Disease as a Kailedoscopic Clinical Spectrum

Diagnosis of the atypical forms of KD remains challenging for most clinicians and pediatricians. According to the AHA recommendations, atypical KD is considered in children with prolonged fever and at least two compatible classic findings of KD associated with echocardiographic findings of CAA, abnormal labwork signs, and different non-canonical manifestations not included in the KD diagnostic clues. Our narrative review consisted of selecting articles published until December 2022 dealing with case reports or cohorts of children who matched the definition of ‘atypical’ KD. All results have been tabulated and analyzed in Table 1 and Table 2. The first one reports on the general data and canonical KD manifestations found in such case reports with atypical KD; the second one details the non-canonical manifestations which were observed in these same patients, challenging the proper diagnosis of KD [25,26,27,28,29,30,31,32,33,34,35,36,37,38,39,40,41,42,43,44,45,46,47,48,49,50,51,52,53,54,55,56,57,58,59,60,61,62,63,64,65,66,67,68,69,70,71,72,73,74,75,76,77,78,79,80,81,82].

Atypical KD was more prevalent in males (1.85:1; 65% versus 35%); patients’ age in the case reports analyzed fell into the interval of 2 months–14 years. In many cases the disease started in the first 6 months of life. Most authors observed that the occurrence of CAA in patients with atypical KD was related to a multitude of nonspecific clinical manifestations, shading the correct diagnostic path and leading to delayed recognition of KD and delayed start of therapy. A retrospective study in Indian children with KD showed that no specific clinical differences regarding age could be found in their cohort, but atypical forms of KD were associated with longer hospitalizations with no difference in the relationship with long-term CAA outcomes [26]. Myocardial infarction related to multiple coronary artery aneurysms leading to cardiovascular collapse was also observed; autoptical findings revealed that mononuclear cell infiltration was predominant in the coronary arterial walls of these patients [27]. Table 3 shows the different types of coronary artery involvement reported in patients with atypical KD who were evaluated in our analysis.

Coronary arteries were the most common location for aneurysms in patients with atypical KD, but also the involvement of systemic vessels had been disclosed. As reported by Kantaci et al., several patients with atypical KD presented vascular lesions which were misdiagnosed by ultrasound assessment or magnetic resonance angiography, but were detected by high-pitch low-dose CT angiography [28].

Pulmonary and gastrointestinal symptoms were the most frequently found atypical features of KD in our review. Gastrointestinal acute symptoms related to atypical KD included vomiting, diarrhea, and abdominal pain, but also hepatomegaly, cholestatic hepatitis, pancreatitis, and hydrops of the gallbladder [29,30]. Stoler et al. suggested considering KD-related pancreatitis in the differential diagnosis of febrile patients with severe abdominal symptoms [31]. Intestinal pseudo-obstruction has been also described [32,33,34]. The pathogenesis of gut pseudo-obstruction is not clear, although vasculitis occurring in the mesenteric artery might lead to ischemia and dysfunction of the myenteric plexus [35]. Antibiotic-resistant pneumonia can herald atypical KD, but a peculiar retropharyngeal involvement with abscess-like lesions may also be an infrequent manifestation of atypical KD [36,37,38,39,40]. Renal involvement includes interstitial nephritis, hemolytic uremic syndrome, immune complex-mediated nephropathy, and acute nephritic syndrome. Nephrotic syndrome is rarely found as KD atypical manifestation, but the most frequently found innocent renal sign of KD is sterile pyuria, reported in up to 80% of cases [41,42,43].

The incidence of pulmonary manifestations in KD was 1.83% in a retrospective cohort of 602 patients described by Singh et al. All patients were analyzed with X-ray chest films and ultrasound investigations, which largely revealed pulmonary consolidations [44]. Moreover, 63.6% of these patients had no clinical features, which suggests a diagnosis of KD either on their history or at presentation. Pleural and interstitial lung involvement has been also reported as atypical clues of KD [45,46]. As reported by Yasukawa et al., pulmonary involvement in KD may be due to the upregulation of the vascular endothelial growth factor in lung and heart vessels, leading to increased vascular permeability and perivascular inflammatory changes [47].

Central nervous system involvement in KD can be proved by different signs occurring at disease onset, such as aseptic meningitis, ataxia, seizures, facial or other peripheral nerve palsies, hemiparesis, and/or lethargy [48,49]. As reported by Zhang et al., many atypical KD manifestations may be a consequence of the vascular leak associated with KD, leading to extravascular leakage of plasma into different districts [50,51]. Regarding the skin, a frequently reported KD-related manifestation is hardening and erythema at the scar of the Calmette–Guérin vaccine administration site [52]. Hemophagocytic lymphohistiocytosis is a dreadful systemic picture of prolonged fever and hyperinflammation overlapping with KD symptoms, described in a few patients with atypical KD [53,54,55]. KD-related shock syndrome represents a severe form of multi-organ failure and hemodynamic instability occurring in atypical KD, which is also characterized by a higher rate of IVIG failure [56,57,58,59,60].

As for labwork abnormalities in atypical KD, increased CRP, increased erythrocyte sedimentation rate, anemia, neutrophil leukocytosis, hypoalbuminemia, and hypertransaminasemia can be present in 25.4% of patients with atypical disease manifestations [61]. In addition, the role of the N-terminal pro-brain natriuretic peptide (NT-proBNP), produced by the myocardium, might have a diagnostic contribution in identifying KD patients from children with protracted undifferentiated febrile illnesses and establish a timely diagnosis for atypical KD [62]. Prospective larger cohort studies are needed to help determine the best cut-off values and further clarify the role of NT-proBNP in KD.

## 4. Refractoriness to Intravenous Immunoglobulin in Kawasaki Disease as an Ultimate Challenge to Clinicians

About 10–20% of patients with KD are unresponsive to IVIG, requiring secondary treatments after standard therapy, and are at increased risk of CAA. Their early identification should be critical to initiate IVIG with further additional therapies, but the available scoring systems are poorly sensitive in non-Japanese populations [22,23]. Many attempts to develop universal scoring systems and detect children at higher risk of IVIG resistance have been unsuccessful, and risk scores in use have not been fully validated, with the exception of the Kobayashi score (consisting of patient’s age, neutrophil count, platelet count, CRP, sodium, aspartate transaminase, and days of illness prior to IVIG infusion) [22,23]. Moreover, the definition of “IVIG resistance” based on the persistence or recrudescence of fever (≥38 °C) after the completion of IVIG infusion is not homogenous, as the period of observation may vary from 36 h to 7 days. Furthermore, fever alone may not be the unique indicator of an insufficient response to IVIG, as the persistence of high inflammatory markers (i.e., elevated CRP) might reflect a higher risk of diffuse hyper-inflammation and subsequent risk of cardiac complications.

A lot of studies have attempted to discriminate between KD and other febrile illnesses based on certain laboratory parameters, but the major shortcoming has been the selection of febrile controls, which might not represent the population of patients who could be confused with KD patients. Further limitations of these studies include selection biases, as the potentially most severe patients hospitalized in intensive care units were not assessed and some subjects had insufficient data to calculate a score (thus reducing the overall statistical power of conclusions). Moreover, the use of different models for the prediction of IVIG resistance in Japanese children may not be sufficiently accurate and sensitive in other populations.

A retrospective study recruiting 1953 KD patients admitted to Shanghai hospitals between 1998 and 2007 identified clinical and laboratory parameters as potential predictors of IVIG resistance at its first infusion, which were: (a) lymph node enlargement, (b) platelet count ≥ 530,000/mm^3^, and (c) erythrocyte sedimentation rate ≥75 mm/h [83]. However, this study had limitations with regard to both pediatric populations studied (only patients from Shanghai) and differences in IVIG resistance between KD patients with classic and atypical presentations. Another retrospective study carried out in a single center (Korea’s largest hospital in Seoul) evaluated the presence of clinical laboratory predictors of non-responsiveness to a second infusion of IVIG in 588 KD patients. White blood cell count, neutrophil count, platelet count, hemoglobin, serum protein level, albumin, potassium, and CRP were all found statistically significant predictors of resistance to a second cycle of IVIG at the univariate analysis; however, serum protein level was the only statistically significant predictor to a second cycle of IVIG at the multivariate analysis [84]. An extensive multicenter study by Kim et al. included 5151 patients with KD, and 524 of them (10.2%) were non-responders to IVIG. Laboratory data showed significant differences between the IVIG-responsive and IVIG-resistant groups, as the second one had a higher level of CRP levels, neutrophil count, transaminases, and NT-proBNP. The multivariate logistic regression analysis between KD patients with or without CAA, both responder and resistant to IVIG, revealed that neutrophil count, CRP, transaminase, and NT-proBNP levels were significantly higher in the IVIG-resistant group, while albumin was significantly lower; only CRP was significantly associated to a higher occurrence of CAA, while neutrophil count, transaminases, NT-proBNP, and albumin were not [85]. The RAISE study carried out in Japan, involving KD patients at higher risk of IVIG resistance based on the Kobayashi score, used a double treatment approach in a double-blind randomized fashion in which a subgroup was subjected to IVIG combined with prednisolone (2 mg/kg/day for 5 days) and the other with a placebo in addition to IVIG. The results of the study showed a reduction in fever duration and a better reduction in the coronary artery *z*-score for KD patients treated with add-on corticosteroids [86]. Furthermore, Do et al. found that a relatively higher proportion of neutrophils and a lower proportion of lymphocytes during the KD subacute phase could predict IVIG-resistant cases [87]. Sleeper et al. evaluated three Japanese risk scoring systems for IVIG resistance developed in Japan, finding that they had low sensitivity (<45%) but good specificity if applied to an independent dataset of North American children of mixed ethnicities. The same authors examined whether the risk scores for IVIG resistance were correlated with the outcome of coronary artery dimensions, finding no significant relationship [88]. Athappan et al. evaluated four randomized trials dealing with primary corticosteroid treatment in addition to IVIG in patients with KD, suggesting that such treatment could decrease the rate of IVIG re-treatment, although it was shown to lower neither the risk of coronary artery aneurysms, nor the risk of any cardiovascular adverse events [89]. Piram et al. recently tried to build a new scoring system to predict IVIG unresponsiveness for children with KD in a large multi-ethnic French population, finding that predictors of secondary treatment with IVIG after a first IVIG infusion were hepatomegaly, increased level of alanine aminotransferase, low lymphocyte count (<2400/mm^3^), and shortness of time before starting IVIG since disease onset [90]. Labwork abnormalities might provide helpful information, but no study has ever validated the clinical use of any laboratory abnormality to guide critical decisions for KD management [91,92].

## 5. Is There Any Peculiar Biologic Cue in Refractory Children with Kawasaki Disease?

The prediction of IVIG resistance in children with KD should be crucial to intensify the initial treatment, aiming to prevent cardiac complications. In particular, KD with atypical presentations remains the paramount challenge for most physicians, as KD might remain undiagnosed for many days and lose the opportunity for prompt treatment. Although the pathogenetic mechanism underlying KD is not yet clearly known, some genetic studies have suggested a possible upregulation of the NFAT (nuclear T-cell activation factor), which is involved in stimulating the T-cell machinery in refractory cases. For this reason, the role of cyclosporine as a calcineurin inhibitor involved in the NFAT pathway has been advocated to manage the complex cases of KD. In particular, a randomized double-blind trial evaluating 175 KD patients at higher risk for IVIG resistance via Kobayashi score considered a first subgroup treated with cyclosporine, in addition to IVIG, and a second one was treated with a placebo. As a result, the subgroup treated with cyclosporine showed a lower incidence of CAA than the other [19].

KD may show a changeable evolution and different response patterns according to unraveled factors, which may be of demographic, genetic, or epigenetic nature. Based on the large and positive experience with TNF-inhibitors in autoinflammatory disorders [93,94,95,96], an opportunity has emerged to use etanercept as an anti-TNF strategy to treat IVIG-resistant patients with KD, although its clinical efficacy has been higher in older patients (over 1 year of age) [21]. There are also anecdotal experiences of severe cases of atypical KD refractory to IVIG who were successfully treated with infliximab or anakinra. Their effectiveness as adjunctive therapies in non-responder KD has been highlighted by different reports [20,56,97]. Information on the role of additional treatments to IVIG with cyclosporine, etanercept, infliximab, or anakinra awaits the results of ongoing trials. Future identification of novel biomarkers and factors involved in host predisposition to cardiovascular risks may further help to personalize therapy in KD.

## 6. Conclusions

Since the initial description of KD many dilemmas about this mysterious disorder and its management remain outstanding. However, without a definitive cause recognized and without a reliable gold-standard diagnostic test, the exact identification of KD and the decision to initiate therapy depend solely on its clinical scenario and on the pediatrician’s perspicacity. Most clinicians use the term “atypical” KD to describe children who fail to meet the case definition for classic KD and display non-canonical manifestations, mostly if they have compatible laboratory findings and no other explanation for their illness; in these cases, the diagnosis of KD can be fully confirmed when overt CAA are identified by echocardiography. However, coronary artery dilatations are generally not detected until after the first week of illness, and a normal echocardiogram in the first week of illness could not rule out the diagnosis of KD [98,99].

Our narrative review aimed at evaluating case reports of children with atypical KD, which provided substantial information to readers, as well as studies assessing non-responsiveness to IVIG in KD. All case reports containing the words “SARS-CoV-2”, “COVID”, and “MIS-C” were excluded by this revision. We found that atypical KD has been more represented in males and that the overall age interval for these patients was 2 months–14 years. Our analysis has shown that atypical pulmonary or gastrointestinal manifestations, but also neurological manifestations, in a febrile child with raised inflammatory markers should prompt the clinician to think of KD. A non-negligible percentage of patients, especially in the first 6 months of life, might have atypical forms of KD, whose painstaking differential diagnosis with infectious diseases displaying signs of mucocutaneous inflammation, which closely mimic KD, can be insidious. The integration of clinical data, labwork findings and instrumental results remains recommended, but having a high index of suspicion among persistently febrile children is crucial. This integrated approach usually allows for starting appropriate therapy with IVIG and, hopefully, for decreasing the occurrence of CAA. Failure to respond to IVIG in KD is another relevant issue, still puzzling for these children who remain at a higher risk of cardiovascular complications. About 10–20% of patients with KD are unresponsive to IVIG and will require secondary treatments after standard IVIG. The prediction of IVIG resistance should be crucial to intensify the initial treatment, but no scores can be universally used today for this aim. Further research is needed to elucidate all open questions about this complex disease and clarify the long-term outcome of its cardiovascular complications.

## Figures and Tables

**Table 1:** List of the general features and classical manifestations (according to the American Heart Association criteria) in case reports presenting ‘atypical’ pictures of Kawasaki disease.

	*Reference*	Age (Months)	Gender	Fever (>7 Days)	Rash	Oral Changes	Bulbar Signs	Cervical Adenitis	Extremity Changes
1	Ramamoorthy et al. [45]	19	M	YES	NO	NO	NO	NO	NO
2	Prokic et al. [30]	72	M	YES	YES	NO	NO	NO	NO
3	Rosario et al. [63]	2	M	YES	NO	NO	NO	YES	NO
4	Cason et al. [36]	168	M	YES	NO	NO	NO	YES	NO
5	Chaudhuri et al. [64]	5	M	YES	NO	NO	YES	NO	NO
6	Dyer et al. [65]	72	M	NO	NO	NO	YES	NO	NO
7	de Magalhães et al. [66]	3	F	YES	YES	NO	NO	NO	NO
8	Singh et al. [67]	102	M	NO	NO	NO	NO	NO	NO
9	D’Auria et al. [68]	48	M	YES	NO	NO	NO	YES	NO
10	Micallef et al. [69]	9	M	NO	NO	NO	NO	NO	NO
11	Tiao et al. [32]	30	M	YES	NO	NO	NO	NO	YES
12	Thapa et al. [70]	7	M	YES	NO	NO	NO	NO	NO
13	Peduzzi et al. [25]	3	F	NO	YES	NO	NO	NO	NO
14	Kim et al. [37]	108	F	NO	YES	YES	NO	YES	YES
15	Papadodima et al. [41]	132	M	YES	NO	NO	NO	NO	YES
16	Ren et al. [71]	12	M	YES	YES	NO	NO	NO	NO
17	Catalano-Pons et al. [49]	3	M	YES	NO	NO	YES	NO	NO
18	Sahoo et al. [72]	31	M	YES	NO	NO	NO	NO	YES
19	Sahoo et al. [72]	56	F	YES	YES	NO	NO	NO	NO
20	Sahoo et al. [72]	60	M	YES	NO	NO	NO	NO	YES
21	Behjati-Ardakani et al. [73]	120	F	YES	NO	NO	NO	NO	NO
22	Usta Guc et al. [74]	9	F	YES	NO	NO	YES	NO	NO
23	Godart et al. [33]	7	F	YES	NO	NO	YES	NO	NO
24	O’Byrne et al. [75]	8	M	YES	YES	NO	NO	NO	NO
25	Kritsaneepaiboon et al. [38]	10	F	YES	NO	NO	NO	NO	YES
26	Torres et al. [76]	31	M	YES	NO	NO	YES	NO	NO
27	Uziel et al. [46]	30	F	YES	NO	NO	NO	NO	YES
28	Uziel et al. [46]	24	F	YES	NO	NO	NO	NO	NO
29	Yang et al. [77]	48	M	YES	NO	NO	NO	NO	NO
30	Doğan et al. [78]	4	F	YES	NO	NO	NO	NO	NO
31	Guile et al. [79]	3	M	YES	YES	NO	NO	NO	NO
32	Pinches et al. [80]	3	M	NO	NO	NO	NO	NO	NO
33	Uchida et al. [81]	20	M	YES	NO	NO	YES	NO	NO
34	Choi et al. [82]	12	F	NO	NO	NO	NO	NO	NO

**Table 2:** List of the non-canonical findings in case reports presenting ‘atypical’ pictures of Kawasaki disease.

	*Reference*	Pulmonary Signs	Gastrointestinal Symptoms	Kidney Involvement	Other Involved Organs
1	Ramamoorthy et al. [45]	Pneumonia, respiratory distress	NO	NO	NO
2	Prokic et al. [30]	NO	Abdominal pain, hydrops of the gallbladder, pancreatitis	NO	NO
3	Rosario et al. [63]	NO	NO	NO	Pericardial effusion with shock
4	Cason et al. [36]	NO	NO	NO	Retropharyngeal abscess
5	Chaudhuri et al. [64]	NO	NO	NO	NO
6	Dyer et al. [65]	NO	NO	NO	Torticollis
7	de Magalhães et al. [66]	Pulmonary thrombosis	NO	NO	BCG scar erythema, systemic and pulmonary thrombosis, dilatation of iliac arteries
8	Singh et al. [67]	Respiratory distress, pleural effusion	Vomiting, nausea, abdominal pain	NO	Dilatation of systemic arteries with shock
9	D’Auria et al. [68]	NO	Vomiting, diarrhoea	NO	Exudative pericarditis, anasarca
10	Micallef et al. [69]	Tachypnoea	Vomiting	NO	Urticaria
11	Tiao et al. [32]	NO	Small bowel obstruction, vomiting with coffee-ground features	NO	NO
12	Thapa et al. [70]	NO	NO	NO	NO
13	Peduzzi et al. [25]	Tachypnoea	NO	NO	Pericardial effusion, myocardial infarction with shock
14	Kim et al. [37]	NO	NO	NO	Retropharyngeal abscess
15	Papadodima et al. [41]	NO	Melena	Hematuria	Pericardial effusion, coronary artery thrombosis
16	Ren et al. [71]	NO	NO	NO	NO
17	Catalano-Pons et al. [49]	NO	NO	NO	Aseptic meningitis
18	Sahoo et al. [72]	Respiratory distress	Vomiting, abdominal pain	NO	Congestive heart failure
19	Sahoo et al. [72]	Respiratory distress	NO	NO	Congestive heart failure
20	Sahoo et al. [72]	Respiratory distress	NO	NO	Congestive heart failure
21	Behjati-Ardakani et al. [73]	NO	NO	NO	Wrist arthritis, arthralgia of hips, knees and ankles, neck pain
22	Usta Guc et al. [74]	NO	NO	NO	Severe irritability
23	Godart et al. [33]	NO	Abdominal obstruction, necrosis of the gut, short-bowel syndrome	NO	NO
24	O’Byrne et al. [75]	Respiratory distress	Melena	NO	Pericardial effusion, coronary artery thrombosis
25	Kritsaneepaiboon et al. [38]	NO	NO	NO	Retropharyngeal abscess, brightening, cuffing or wall irregularities of coronary arteries
26	Torres et al. [76]	NO	NO	NO	NO
27	Uziel Y et al. [46]	Pulmonary consolidation	NO	NO	Bullous meningitis
28	Uziel et al. [46]	Pulmonary consolidation	Vomiting	NO	Anasarca
29	Yang et al. [77]	NO	Abdominal pain, hydrops of the gallbladder, pancreatitis	NO	Cardiac arrest
30	Doğan et al. [78]	NO	NO	NO	Myocardial infarction
31	Guile et al. [79]	NO	Gallbladder	NO	Pericardial effusion
32	Pinches et al. [80]	NO	NO	NO	Heart murmur
33	Uchida et al. [81]	NO	NO	NO	Coronary artery aneurysm
34	Choi et al. [82]	NO	NO	NO	Heart murmur

**Table 3:** Specific types of coronary artery involvement in case reports presenting ‘atypical’ pictures of Kawasaki disease.

	*Reference*	CA Dilatations	Small CAA	Average CAA	Large or Giant CAA
1	Ramamoorthy et al. [45]	YES (LAD)	NO	YES (LMCA)	NO
2	Prokic et al. [30]	NO	NO	NO	NO
3	Rosario et al. [63]	YES	NO	NO	NO
4	Cason et al. [36]	NO	NO	NO	YES
5	Chaudhuri et al. [64]	YES (LAD)	YES (LMCA)	NO	NO
6	Dyer et al. [65]	NO	NO	NO	YES (LMCA, RCA)
7	de Magalhães et al. [66]	NO	NO	YES (LMCA, RCA)	NO
8	Singh et al. [67]	NO	NO	YES (LMCA, RCA)	YES (LAD)
9	D’Auria et al. [68]	NO	YES (RCA)	NO	NO
10	Micallef et al. [69]	NO	YES	NO	YES
11	Tiao et al. [32]	YES	NO	NO	NO
12	Thapa et al. [70]	NO	NO	YES (LAD)	NO
13	Peduzzi et al. [25]	NO	YES	NO	NO
14	Kim et al. [37]	NO	NO	NO	NO
15	Papadodima et al. [41]	NO	NO	NO	NO
16	Ren et al. [71]	YES	YES	NO	NO
17	Catalano-Pons et al. [49]	YES	NO	YES (LMCA)	NO
18	Sahoo et al. [72]	NO	NO	NO	YES
19	Sahoo et al. [72]	NO	NO	NO	YES
20	Sahoo et al. [72]	NO	NO	NO	YES (LAD)
21	Behjati-Ardakani et al. [73]	NO	NO	NO	YES (RCA, LMCA)
22	Usta Guc et al. [74]	YES (RCA, LMCA)	NO	NO	NO
23	Godart et al. [33]	NO	NO	NO	YES
24	O’Byrne et al. [75]	NO	NO	NO	YES (RCA, LMCA)
25	Kritsaneepaiboon et al. [38]	NO	NO	NO	NO
26	Torres et al. [76]	NO	NO	NO	YES (LMCA)
27	Uziel et al. [46]	NO	NO	NO	NO
28	Uziel et al. [46]	NO	NO	NO	YES
29	Yang et al. [77]	YES (RCA)	NO	NO	YES (LAD)
30	Doğan et al. [78]	YES (RCA, LMCA)	NO	NO	NO
31	Guile et al. [79]	NO	NO	NO	YES (RCA, LMCA)
32	Pinches et al. [80]	NO	NO	YES (LAD)	NO
33	Uchida et al. [81]	YES	NO	NO	NO
34	Choi et al. [82]	NO	YES (LAD)	NO	NO

CA = coronary artery; CAA = coronary artery abnormalities; LAD = left anterior descending artery; LMCA = left main coronary artery; RCA = right coronary artery.

## Data Availability

Not applicable.

## References

[1] Nakamura Y. (2018). Kawasaki disease: Epidemiology and the lessons from it. Int. J. Rheum. Dis..

[2] McCrindle B.W., Rowley A.H., Newburger J.W., Burns J.C., Bolger A.F., Gewitz M., Baker A.L., Jackson M.A., Takahashi M., Shah P.B. (2017). Diagnosis, treatment, and long-term management of Kawasaki disease: A scientific statement for health professionals from the American Heart Association. Circulation.

[3] Marchesi A., Tarissi de Jacobis I., Rigante D., Rimini A., Malorni W., Corsello G., Bossi G., Buonuomo S., Cardinale F., Cortis E. (2018). Kawasaki disease: Guidelines of the Italian Society of Pediatrics, part I-definition, epidemiology, etiopathogenesis, clinical expression and management of the acute phase. Ital. J. Pediatr..

[4] Marchesi A., Tarissi de Jacobis I., Rigante D., Rimini A., Malorni W., Corsello G., Bossi G., Buonuomo S., Cardinale F., Cortis E. (2018). Kawasaki disease: Guidelines of Italian Society of Pediatrics, part II-treatment of resistant forms and cardiovascular complications, follow-up, lifestyle and prevention of cardiovascular risks. Ital. J. Pediatr..

[5] Marchesi A., Rigante D., Cimaz R., Ravelli A., Tarissi de Jacobis I., Rimini A., Cardinale F., Cattalini M., De Zorzi A., Dellepiane R.M. (2021). Revised recommendations of the Italian Society of Pediatrics about the general management of Kawasaki disease. Ital. J. Pediatr..

[6] Kim G.B. (2019). Reality of Kawasaki disease epidemiology. Korean J. Pediatr..

[7] Mauro A., Fabi M., Da Frè M., Guastaroba P., Corinaldesi E., Calabri G.B., Giani T., Simonini G., Rusconi F., Cimaz R. (2016). Kawasaki disease: An epidemiological study in central Italy. Pediatr. Rheumatol..

[8] Cimaz R., Fanti E., Mauro A., Voller F., Rusconi F. (2017). Epidemiology of Kawasaki disease in Italy: Surveillance from national hospitalization records. Eur. J. Pediatr..

[9] Rigante D. (2020). Kawasaki disease as the immune-mediated echo of a viral infection. Mediterr. J. Hematol. Infect. Dis..

[10] Manna R., Rigante D. (2021). The everchanging framework of autoinflammation. Intern. Emerg. Med..

[11] Sangiorgi E., Rigante D. (2022). The clinical chameleon of autoinflammatory diseases in children. Cells.

[12] Rigante D., Frediani B., Galeazzi M., Cantarini L. (2013). From the Mediterranean to the sea of Japan: The transcontinental odyssey of autoinflammatory diseases. Biomed. Res. Int..

[13] Rigante D. (2017). A systematic approach to autoinflammatory syndromes: A spelling booklet for the beginner. Expert. Rev. Clin. Immunol..

[14] Rajasekaran K., Duraiyarasan S., Adefuye M., Manjunatha N., Ganduri V. (2022). Kawasaki disease and coronary artery involvement: A narrative review. Cureus.

[15] Onouchi Z., Tomizawa N., Goto M., Nakata K., Fukuda M., Goto M. (1975). Cardiac involvement and prognosis in acute mucocutaneous lymph node syndrome. Chest.

[16] Li D., Chen X., Li X., Yuan Y., Jin H., Liu G., Zhang H., Xie G. (2021). Effectiveness and safety of dual antiplatelet therapy in coronary aneurysms caused by Kawasaki disease in children: Study protocol for a multicenter randomized clinical trial. Transl. Pediatr..

[17] de La Harpe M., Di Bernardo S., Hofer M., Sekarski N. (2019). Thirty years of Kawasaki disease: A single-center study at the University Hospital of Lausanne. Front. Pediatr..

[18] Rigante D., Valentini P., Rizzo D., Leo A., De Rosa G., Onesimo R., De Nisco A., Angelone D.F., Compagnone A., Delogu A.B. (2010). Responsiveness to intravenous immunoglobulins and occurrence of coronary artery abnormalities in a single-center cohort of Italian patients with Kawasaki syndrome. Rheumatol. Int..

[19] Hamada H., Suzuki H., Onouchi Y., Ebata R., Terai M., Fuse S., Okajima Y., Kurotobi S., Hirai K., Soga T. (2019). Efficacy of primary treatment with immunoglobulin plus ciclosporin for prevention of coronary artery abnormalities in patients with Kawasaki disease predicted to be at increased risk of non-response to intravenous immunoglobulin (KAICA): A randomised controlled, open-label, blinded-endpoints, phase 3 trial. Lancet.

[20] Maggio M.C., Cimaz R., Alaimo A., Comparato C., Di Lisi D., Corsello G. (2019). Kawasaki disease triggered by parvovirus infection: An atypical case report of two siblings. J. Med. Case Rep..

[21] Portman M.A., Dahdah N.S., Slee A., Olson A.K., Choueiter N.F., Soriano B.D., Buddhe S., Altman C.A., Del Toro K., Kourtidou S. (2019). Etanercept with IVIG for acute Kawasaki disease: A randomized controlled trial. Pediatrics.

[22] Davies S., Sutton N., Blackstock S., Gormley S., Hoggart C.J., Levin M., Herberg J.A. (2015). Predicting IVIG resistance in UK Kawasaki disease. Arch. Dis. Child..

[23] Rigante D., Andreozzi L., Fastiggi M., Bracci B., Natale M.F., Esposito S. (2016). Critical overview of the risk scoring systems to predict non-responsiveness to intravenous immunoglobulin in Kawasaki syndrome. Int. J. Mol. Sci..

[24] Nomura O., Fukuda S., Ota E., Ono H., Ishiguro A., Kobayashi T. (2021). Monoclonal antibody and anti-cytokine biologics for Kawasaki disease: A systematic review and meta-analysis. Semin. Arthritis Rheum..

[25] Peduzzi T.L., Pitetti R.D. (2002). Myocardial infarction and atypical Kawasaki disease in a 3-month-old infant. Pediatr. Emerg. Care.

[26] Shivalingam G., Prashanth G.P., Hebbal K., Aguiar R. (2017). Clinical presentation and cardiovascular outcome in complete *versus* incomplete Kawasaki disease. Indian. Pediatr..

[27] Yajima D., Shimizu K., Oka K., Asari M., Maseda C., Okuda K., Shiono H., Ohtani S., Ogawa K. (2016). A case of sudden infant death due to incomplete Kawasaki disease. J. Forensic Sci..

[28] Kantarcı M., Güven E., Ceviz N., Oğul H., Sade R. (2019). Vascular imaging findings with high-pitch low-dose dual-source CT in atypical Kawasaki disease. Diagn. Interv. Radiol..

[29] Kılıç B.O., Baysun Ş., Gökşen T.C., Akınbingöl I., Arslan Z. (2018). An unusual presentation of Kawasaki disease: Gallbladder hydrops and acute cholestatic hepatitis. Case Rep. Med..

[30] Prokic D., Ristic G., Paunovic Z., Pasic S. (2010). Pancreatitis and atypical Kawasaki disease. Pediatr. Rheumatol. Online J..

[31] Stoler J., Biller J.A., Grand R.J. (1987). Pancreatitis in Kawasaki disease. Am. J. Dis. Child..

[32] Tiao M.M., Huang L.T., Liang C.D., Ko S.F. (2006). Atypical Kawasaki disease presenting as intestinal pseudo-obstruction. J. Med. Assoc..

[33] Godart F., Bakhti O.M., Bonnevalle M. (2014). Intestinal ischaemia as a severe presentation of Kawasaki disease leading to short-bowel syndrome. Cardiol. Young.

[34] Miyake T., Kawamori J., Yoshida T., Nakano H., Kohno S., Ohba S. (1987). Small bowel pseudo-obstruction in Kawasaki disease. Pediatr. Radiol..

[35] Beiler H.A., Schmidt K.G., von Herbay A., Löffler W., Daum R. (2001). Ischemic small bowel strictures in a case of incomplete Kawasaki disease. J. Pediatr. Surg..

[36] Cason R.K., Ponniah U.P., Makil E.S., Niu M.C. (2020). Atypical recurrent Kawasaki disease with retropharyngeal involvement: A case study and literature review. Ann. Pediatr. Cardiol..

[37] Kim J.S., Kwon S.H. (2016). Atypical Kawasaki disease presenting as a retropharyngeal abscess. Braz. J. Otorhinolaryngol..

[38] Kritsaneepaiboon S., Tanaanantarak P., Roymanee S., Lee E.Y. (2012). Atypical presentation of Kawasaki disease in young infants mimicking a retropharyngeal abscess. Emerg. Radiol..

[39] Isidori C., Sebastiani L., Esposito S. (2019). A case of incomplete and atypical Kawasaki disease presenting with retropharyngeal involvement. Int. J. Env. Res. Public. Health.

[40] Roh K., Lee S.W., Yoo J. (2011). CT analysis of retropharyngeal abnormality in Kawasaki disease. Korean J. Radiol..

[41] Papadodima S.A., Sakelliadis E.I., Goutas N.D., Vlachodimitropoulos D.G., Spiliopoulou C.A. (2009). Atypical Kawasaki disease presenting with symptoms from the genitourinary system: An autopsy report. J. Trop. Pediatr..

[42] Lazea C., Man O., Sur L.M., Serban R., Lazar C. (2019). Unusual presentation of Kawasaki disease with gastrointestinal and renal manifestations. Ther. Clin. Risk Manag..

[43] Chuang G.T., Tsai I., Lin M.T., Chang L.Y. (2016). Acute kidney injury in patients with Kawasaki disease. Pediatr. Res..

[44] Singh S., Gupta A., Jindal A.K., Suri D., Rawat A., Vaidya P.C., Singh M. (2018). Pulmonary presentation of Kawasaki disease-a diagnostic challenge. Pediatr. Pulmonol..

[45] Ramamoorthy J.G., Anantharaj A. (2021). Pulmonary presentation of atypical Kawasaki disease. Indian. J. Pediatr..

[46] Uziel Y., Hashkes P.J., Kassem E., Gottesman G., Wolach B. (2003). “Unresolving pneumonia” as the main manifestation of atypical Kawasaki disease. Arch. Dis. Child..

[47] Yasukawa K., Terai M., Shulman S.T., Toyozaki T., Yajima S., Kohno Y., Rowley A.H. (2002). Systemic production of vascular endothelial growth factor and Fms-like tyrosine kinase-1 receptor in acute Kawasaki disease. Circulation.

[48] Rodriguez-Gonzalez M., Castellano-Martinez A., Perez-Reviriego A.A. (2018). Atypical presentation of incomplete Kawasaki disease: A peripheral facial nerve palsy. J. Emerg. Med..

[49] Catalano-Pons C., Quartier P., Leruez-Ville M., Kaguelidou F., Gendrel D., Lenoir G., Casanova J.L., Bonnet D. (2005). Primary cytomegalovirus infection, atypical Kawasaki disease, and coronary aneurysms in 2 infants. Clin. Infect. Dis..

[50] Zhang Y., Wan H., Du M., Deng H., Fu J., Zhang Y., Wang X., Liu R. (2018). Capillary leak syndrome and aseptic meningitis in a patient with Kawasaki disease: A case report. Medicine.

[51] Saugel B., Umgelter A., Martin F., Phillip V., Schmid R.M., Huber W. (2010). Systemic capillary leak syndrome associated with hypovolemic shock and compartment syndrome. Use of transpulmonary thermodilution technique for volume management. Scand. J. Trauma. Resus Emerg. Med..

[52] Uehara R., Igarashi H., Yashiro M., Nakamura Y., Yanagawa H. (2010). Kawasaki disease patients with redness or crust formation at the bacille Calmette-Guérin inoculation site. Pediatr. Infect. Dis. J..

[53] Stabile A., Bertoni B., Ansuini V., La Torraca I., Sallì A., Rigante D. (2006). The clinical spectrum and treatment options of macrophage activation syndrome in the pediatric age. Eur. Rev. Med. Pharm. Sci..

[54] Latino G.A., Manlhiot C., Yeung R.S., Chanal N., McCrindle B.W. (2010). Macrophage activation syndrome in the acute phase of Kawasaki disease. J. Pediatr. Hematol. Oncol..

[55] Al-Eid W.A., Al-Jefri A., Bahabri S., Al-Mayouf S. (2000). Hemophagocytosis complicating Kawasaki disease. Pediatr. Hematol. Oncol..

[56] Gamez-Gonzalez L.B., Moribe-Quintero I., Castolo M.C., Ortiz J.V., Ramírez M.M., García M.G., Nakashimada M.Y. (2018). Kawasaki disease shock syndrome: Unique and severe subtype of Kawasaki disease. Pediatr. Int..

[57] Kanegaye J.T., Wilder M.S., Molkara D., Frazer J.R., Pancheri J., Tremoulet A.H., Watson V.E., Best B.M., Burns J.C. (2009). Recognition of a Kawasaki disease shock syndrome. Pediatrics.

[58] Dominguez S.R., Friedman K., Seewald R., Anderson M.S., Willis L., Glode M.P. (2008). Kawasaki disease in a pediatric intensive care unit: A case-control study. Pediatrics.

[59] Chen P.S., Chi H., Huang F.Y., Peng C.C., Chen M.R., Chiu N.C. (2015). Clinical manifestations of Kawasaki disease shock syndrome: A case-control study. J. Microbiol. Immunol. Infect..

[60] Gámez-González L.B., Murata C., Muñoz-Ramírez M., Yamazaki-Nakashimada M. (2013). Clinical manifestations associated with Kawasaki disease shock syndrome in Mexican children. Eur. J. Pediatr..

[61] Jun H.O., Yu J.J., Kang S.Y., Seo C.D., Baek J.S., Kim Y.H., Ko J.K. (2015). Diagnostic characteristics of supplemental laboratory criteria for incomplete Kawasaki disease in children with complete Kawasaki disease. Korean J. Pediatr..

[62] Lin K.H., Chang S.S., Yu C.W., Lin S.C., Liu S.C., Chao H.Y., Lee M.T., Wu J.Y., Lee C.C. (2015). Usefulness of natriuretic peptide for the diagnosis of Kawasaki disease: A systematic review and meta-analysis. BMJ Open.

[63] Rosario J.M., Patel F., Levasseur K., Adams L. (2019). Kawasaki at the extremes of age: Thinking outside the box. Pediatr. Emerg. Care.

[64] Chaudhuri M., Jose J., Shenoi A., Tomar M. (2021). Unexpected late-onset aortic valvulitis and moderate regurgitation during longitudinal evaluation of atypical infantile Kawasaki disease: The heart beyond coronaries!. Ann. Pediatr. Cardiol..

[65] Dyer T., Dancey P., Martin J., Shah S. (2018). Torticollis as presentation for atypical Kawasaki disease complicated by giant coronary artery aneurysms. Case Rep. Pediatr..

[66] de Magalhães C.M., Alves N.R., de Melo A.V., Junior C.A., Nόbrega Y.K., Gandolfi L., Pratesi R. (2012). Catastrophic Kawasaki disease unresponsive to IVIG in a 3-month-old infant: A diagnostic and therapeutic challenge. Pediatr. Rheumatol. Online J..

[67] Singh R., Ward C., Walton M., Persad R. (2007). Atypical Kawasaki disease and gastrointestinal manifestations. Paediatr. Child. Health.

[68] D’Auria E., Salvini F., Ruscitto A., Neri I.G., Ballista P., Agostoni C., Riva E. (2009). A case of Kawasaki disease with anasarca and concomitant rotavirus infection. BMJ Case Rep..

[69] Micallef E.S., Attard M.S., Grech V. (2016). A case of atypical Kawasaki disease with giant coronary artery aneurysm containing thrombus. Images Paediatr. Cardiol..

[70] Thapa R., Chakrabartty S. (2009). Atypical Kawasaki disease with remarkable paucity of signs and symptoms. Rheumatol. Int..

[71] Ren Y., Zhang C., Xu X., Yin Y. (2021). A case report of atypical Kawasaki disease presented with severe elevated transaminases and literature review. BMC Infect. Dis..

[72] Sahoo S., Mandal A.K. (2012). Congestive heart failure—An atypical presentation of Kawasaki disease. Iran. J. Pediatr..

[73] Behjati-Ardakani M., Ferdosian F. (2014). Multiple giant saccular and fusiform right and left coronary artery aneurysms after early and adequate treatment of atypical Kawasaki disease with unusual presentation. Acta Med. Iran..

[74] Usta Guc B., Cengiz N., Yildirim S.V., Uslu Y. (2008). Cytomegalovirus infection in a patient with atypical Kawasaki disease. Rheumatol. Int..

[75] O’Byrne M.L., Cohen M.S. (2013). Marked eosinophilia in a patient with history of severe atypical Kawasaki disease. Congenit. Heart Dis..

[76] Torres R.S., Blasco P.B., de la Calzada M.D. (2009). Giant coronary arterial aneurysm in atypical Kawasaki disease. Cardiol. Young.

[77] Yang H., Wang H., Zhang X., Rao L. (2017). Incomplete Kawasaki disease presenting with abdominal pain diagnosed by echocardiography. Anatol. J. Cardiol..

[78] Doğan V., Karaaslan E., Samet Ö.Z., Gümüşer R., Yilmaz R. (2016). Hemophagocytosis in the acute phase of fatal Kawasaki disease in a 4-month-old girl. Balk. Med..

[79] Guile L., Parke S., Kelly A., Tulloh R. (2018). Giant coronary artery aneurysms in a 12-week-old infant with incomplete Kawasaki disease. BMJ Case Rep..

[80] Pinches H., Dobbins K., Cantrell S., May J., Lopreiato J. (2016). Asymptomatic Kawasaki disease in a 3-month-old infant. Pediatrics.

[81] Uchida Y., Iwamoto Y., Urushihara Y., Nagura M., Tanaka R., Arakawa H., Ishido H., Moriwaki K., Masutani S. (2019). Case of incomplete Kawasaki disease with no symptoms except fever causing the development of coronary aneurysm. Int. Heart J..

[82] Choi Y., Eun L.Y., Oh S.H. (2016). Incomplete Kawasaki disease in a 12-month-old girl presenting with cardiac murmur and iron deficiency anemia. Pediatr. Int..

[83] Wei M., Huang M., Chen S., Huang G., Huang M., Qiu D., Guo Z., Jiang J., Zhou X., Yu Q. (2015). A multicenter study of intravenous immunoglobulin non-response in Kawasaki disease. Pediatr. Cardiol..

[84] Seo E., Yu J.J., Jun H.O., Shin E.J., Baek J.S., Kim Y.H., Ko J. (2016). Prediction of unresponsiveness to second intravenous immunoglobulin treatment in patients with Kawasaki disease refractory to initial treatment. Korean J. Pediatr..

[85] Kim M.K., Song M.S., Kim G.B. (2018). Factors predicting resistance to intravenous immunoglobulin treatment and coronary artery lesion in patients with Kawasaki disease: Analysis of the Korean nationwide multicenter survey from 2012 to 2014. Korean Circ. J..

[86] Tremoulet A.H. (2018). Adjunctive therapies in Kawasaki disease. Int. J. Rheum. Dis..

[87] Do Y.S., Kim K.W., Chun J.K., Cha B.H., Namgoong M.K., Lee H.Y. (2010). Predicting factors for refractory Kawasaki disease. Korean Circ. J..

[88] Sleeper L.A., Minich L.L., McCrindle B.M., Li J.S., Mason W., Colan S.D., Atz A.M., Printz B.F., Baker A., Vetter V.L. (2011). Evaluation of Kawasaki disease risk-scoring systems for intravenous immunoglobulin resistance. J. Pediatr..

[89] Athappan G., Gale S., Ponniah T. (2009). Corticosteroid therapy for primary treatment of Kawasaki disease-weight of evidence: A meta-analysis and systematic review of the literature. Cardiovasc. J. Afr..

[90] Federico G., Rigante D., Pugliese A.L., Ranno O., Catania S., Stabile A. (2003). Etanercept induces improvement of arthropathy in chronic infantile neurological cutaneous articular (CINCA) syndrome. Scand. J. Rheumatol..

[91] Rigante D., Lopalco G., Vitale A., Lucherini O.M., De Clemente C., Caso F., Emmi G., Costa L., Silvestri E., Andreozzi L. (2014). Key facts and hot spots on tumor necrosis factor receptor-associated periodic syndrome. Clin. Rheumatol..

[92] Cantarini L., Iacoponi F., Lucherini O.M., Obici L., Brizi M.G., Cimaz R., Rigante D., Benucci M., Sebastiani G.D., Brucato A. (2011). Validation of a diagnostic score for the diagnosis of autoinflammatory diseases in adults. Int. J. Immunopathol. Pharm..

[93] Rigante D. (2010). The protean visage of systemic autoinflammatory syndromes: A challenge for inter-professional collaboration. Eur. Rev. Med. Pharm. Sci..

[94] O’Connor M.J., Saulsbury F.T. (2007). Incomplete and atypical Kawasaki disease in a young infant: Severe, recalcitrant disease responsive to infliximab. Clin. Pediatr..

[95] Barone S.R., Pontrelli L.R., Krilov L.R. (2000). The differentiation of classic Kawasaki disease, atypical Kawasaki disease, and acute adenoviral infection: Use of clinical features and a rapid direct fluorescent antigen test. Arch. Pediatr. Adolesc. Med..

[96] Rigante D. (2009). Autoinflammatory syndromes behind the scenes of recurrent fevers in children. Med. Sci. Monit..

[97] Piram M., Darce Bello M., Tellier S., Di Filippo S., Borealis F., Madhi F., Meinzer U., Cimaz R., Piedvache C., Koné-Paut I. (2020). Defining the risk of first intravenous immunoglobulin unresponsiveness in non-Asian patients with Kawasaki disease. Sci. Rep..

[98] De Rosa G., Pardeo M., Rigante D. (2007). Current recommendations for the pharmacologic therapy in Kawasaki syndrome and management of its cardiovascular complications. Eur. Rev. Med. Pharm. Sci..

[99] Ghelani S.J., Kwatra N.S., Spurney C.F. (2013). Can coronary artery involvement in Kawasaki disease be predicted?. Diagnostics.

